# Dysglycemia in pregnancy and predicted risk of cardiovascular disease 10–14 years after delivery

**DOI:** 10.1002/pmf2.70000

**Published:** 2025-02-25

**Authors:** Kartik K. Venkatesh, William A. Grobman, Jiqiang Wu, Maged M. Costantine, Mark B. Landon, Denise Scholtens, William Lowe, Nilay S. Shah, Sadiya S. Khan

**Affiliations:** ^1^ Department of Obstetrics and Gynecology The Ohio State University Columbus Ohio USA; ^2^ Department of Obstetrics and Gynecology Brown University Providence Rhode Island USA; ^3^ Department of Preventive Medicine Northwestern University Feinberg School of Medicine Chicago Illinois USA; ^4^ Department of Medicine Northwestern University Feinberg School of Medicine Chicago Illinois USA

**Keywords:** cardiovascular disease, cardiovascular health, diabetes, dysglycemia, gestational diabetes, pregnancy, risk, women

## Abstract

We examined whether dysglycemia in the early third trimester of pregnancy was associated with a higher predicted risk of atherosclerotic cardiovascular disease (ASCVD) when measured 10–14 years after delivery. In the prospective Hyperglycemia and Adverse Pregnancy Outcome Follow‐up Study (HAPO FUS) cohort, we found a linear, dose‐response relationship between pregnancy dysglycemia and the predicted long‐term 10‐ and 30‐year risk of ASCVD.

## 1 INTRODUCTION

The frequency of dysglycemia in pregnancy is increasing and gestational diabetes mellitus (GDM) now affects 14% of pregnant individuals globally [1]. Epidemiologic data from nonpregnant individuals demonstrate that higher blood glucose levels increase the risk of atherosclerotic cardiovascular disease (ASCVD) [2], even among individuals without diabetes [3].

The risk of developing ASCVD can be assessed in clinical practice using predictive algorithms, such as the Framingham Risk Score [4], which incorporates multiple cardiometabolic risk factors to calculate an estimate (or risk) of developing ASCVD over the following 10 and 30 years. Such risk estimation can inform the intensity of preventive interventions in the peripartum period by targeting individuals at the highest risk of ASCVD [5].

Prior studies among pregnant individuals have examined the association between clinical diagnoses of dysglycemic states, such as GDM, and ASCVD [6]. However, the association between dysglycemia as a continuous measure in pregnancy without pregestational diabetes and the predicted risk of ASCVD years after delivery but before ASCVD detection has not been well‐examined. This is an important limitation given glycemia and CVD occur along a graded continuum.

We examined whether dysglycemia in the early third trimester was associated with a higher predicted risk of ASCVD when measured 10–14 years after delivery.

## 2 METHODS

We conducted a secondary analysis of pregnant individuals enrolled in the prospective Hyperglycemia and Adverse Pregnancy Outcome Follow‐up Study (HAPO FUS), which was conducted between 2013 and 2016 at 10 international sites [7]. The primary aim of the HAPO FUS was to examine the relationship between pregnancy dysglycemia including GDM and cardiometabolic health 10–14 years after delivery. Key eligibility criteria were: remaining blind to the results of the oral glucose tolerance test (OGTT), having had a gestational age at delivery of ≥ 37 weeks, and not having a diagnosis of pregestational diabetes.

The exposure was blood glucose defined by a sum of *z*‐scores across the three timepoints incorporating the fasting, 1‐h, and 2‐h values of a 75‐g OGTT at ∼28 weeks’ gestation; and, second, each *z*‐score at the three timepoints assessed separately [7]. Participants and providers remained blinded to OGTT results and participants did not receive treatment for GDM. Consistent with prior HAPO analyses, this variable was created by calculating a summary *z* scores by subtracting the appropriate mean from each individual's glucose measurements, dividing by the corresponding SD, and then summing the three resulting *z* scores for each individual [8]. This standardized measure allowed for comparison of glucose levels across different individuals within the study.

The outcomes were the predicted 10‐year and 30‐year risk of ASCVD (a composite of fatal and nonfatal coronary heart disease and stroke) using the Framingham Risk Score (https://www.framinghamheartstudy.org/fhs‐risk‐functions/cardiovascular‐disease‐30‐year‐risk/) assessed at 10–14 years after delivery [4]. This risk score is a multivariable function that includes eight cardiometabolic risk factors (age, sex, total cholesterol, high density lipoprotein cholesterol, smoking status, systolic blood pressure, hypertension treated with medication, and diabetes) to calculate the probability of developing incident ASCVD over the next 10 or 30 years [4]. The Framingham Risk Score was calculated for this secondary analysis and was employed because it has been previously used among individuals of reproductive age and across international settings [9].

The continuous relationship between glucose and CVD risk was visually modelled using Lowess curves or weighted linear least squares regression. Linear regression models adjusted for covariates measured at the baseline pregnancy visit when the OGTT was performed including study field center, age, body mass index, smoking and alcohol use, parity, and time from delivery to postpartum assessment.

## 3 RESULTS

Among 4545 assessed pregnant individuals, the median age was 30.6 years and the median gestational age was 27.9 weeks at enrollment. The median BMI was 26.6 kg/m^2^. Over half (51.2%) were parous.

**FIGURE 1:**
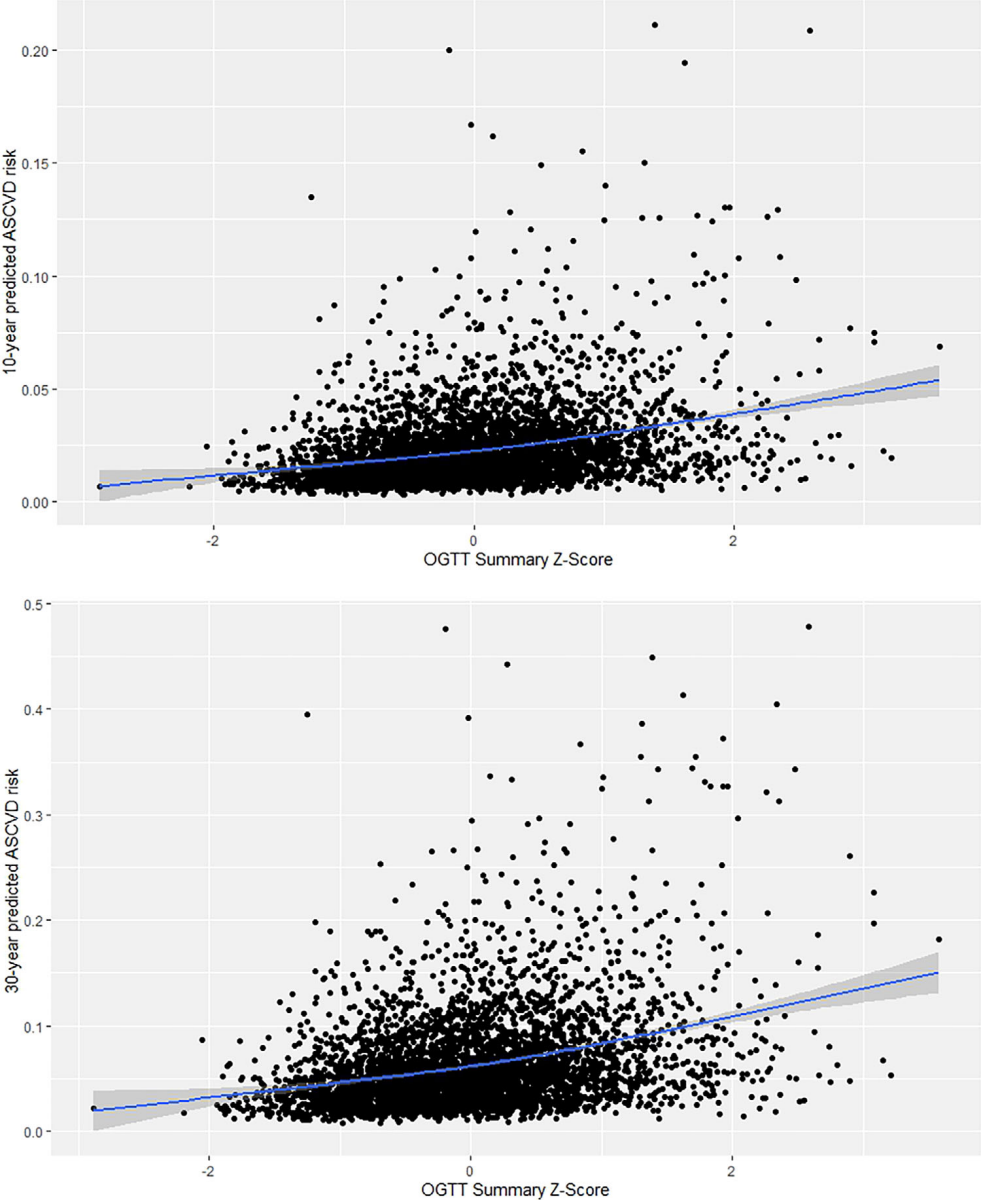
(A and B) Relationship glucose *z*‐score in the early third trimester and estimated 10‐ and 30‐year ASCVD risk.

At 10–14 years after delivery, at a median age of 41.1 years, the median predicted 10‐year risk of ASCVD was 1.8% (IQR: 1.2%, 2.7%) and 30‐year risk was 5.0% (IQR: 3.3%, 7.7%). Lowess curves, which display the fitted line with 95% confidence intervals (95% CI), demonstrate the association between higher glucose *z*‐score values and estimated ASCVD risk over the 10‐ and 30‐year periods (Figure 1A,B).

In adjusted analyses, for each 1‐unit higher overall glucose *z*‐score (or one standard deviation [SD] above the mean), the estimated 10‐year ASCVD risk was higher by 0.30% (95% CI: 0.23 to 0.37), and the estimated 30‐year ASCVD risk was higher by 0.89% (95% CI: 0.70 to 1.08) (Table 1).

**TABLE 1:** Association between pregnancy dysglycemia and predicted 10‐ and 30‐year risk of ASCVD postpartum.

	Unadjusted absolute difference in ASCVD risk (95% CI)	Adjusted absolute difference in ASCVD risk (95% CI)
**10‐year predicted ASCVD risk**		
1 unit increase in overall glucose *z*‐score	0.69 (0.60, 0.77)	0.30 (0.23, 0.37)
1 unit increase in fasting glucose *z*‐score	0.41 (0.34, 0.47)	0.14 (0.08, 0.19)
1 unit increase in 1‐h glucose *z*‐score	0.45 (0.39, 0.51)	0.21 (0.15, 0.26)
1 unit increase in 2‐h glucose *z*‐score	0.42 (0.36, 0.48)	0.20 (0.15, 0.25)
**30‐year predicted ASCVD risk**		
1 unit increase in overall glucose *z*‐score	1.95 (1.72, 2.17)	0.89 (0.70, 1.08)
1 unit increase in fasting glucose *z*‐score	1.15 (0.99, 1.32)	0.43 (0.29, 0.57)
1 unit increase in 1‐h glucose *z*‐score	1.28 (1.11, 1.44)	0.58 (0.45, 0.72)
1 unit increase in 2‐h glucose *z*‐score	1.19 (0.09, 1.36)	0.57 (0.44, 0.71)

^a^ Linear regression was used.

^b^ Models adjusted for variables at oral glucose tolerance test during pregnancy: study field center, age (continuous), BMI (continuous), smoking status (yes/no), alcohol use (yes/no), parity (0, > 1), and time from delivery to postpartum assessment (continuous).

Abbreviation: atherosclerotic cardiovascular disease (ASCVD).

In secondary analyses, these adjusted associations between glucose *z*‐score and 10‐ and 30‐year predicted risk of ASCVD persisted when assessed separately at fasting, 1‐h, and 2‐h timepoints (Table 1).

## 4 DISCUSSION

In the HAPO FUS prospective cohort, we found a linear, dose‐response relationship between dysglycemia identified in the early third trimester and the predicted 10‐ and 30‐year risk of ASCVD measured 10–14 years after delivery.

The association of an increase in the overall or summary glucose level of 1 SD in pregnancy and predicted ASCVD risk builds on findings from the original HAPO cohort that found the risk of adverse pregnancy outcomes associated with an increase in the fasting glucose level of 1 SD (6.9 mg per deciliter), an increase in the 1‐h glucose level of 1 SD (30.9 mg per deciliter), and an increase in the 2‐h glucose level of 1 SD (23.5 mg per deciliter) [10]. In addition, these findings are consistent with prior analyses from the HAPO FUS that have found a continuous association between pregnancy dysglycemia and individual postpartum cardiometabolic outcomes, including diabetes, hypertension, obesity, and hyperlipidemia [7], but not a composite measure of future CVD risk as used in the current study.

The current study measured estimated ASCVD risk based on a composite of several atherogenic cardiometabolic risk factors. These findings are consistent with prior data from non‐pregnant individuals that have found that blood glucose is continuously related to CVD risk [2, 3].

Further research is needed to better understand how different glucose patterns in pregnancy, particularly among those without a diagnosis of GDM, are associated with long‐term CVD risk. Investigation of continuous glucose monitoring in pregnancy may facilitate identification of glucose patterns associated with an increased risk of adverse long‐term cardiovascular health. These results emphasize the ability to identify individuals at a higher risk of ASCVD using pregnancy data [11]. Continued risk assessment and engagement in preventive care in the peripartum period is an opportunity for cardiovascular health promotion [12]. Individuals deemed to be at increased risk could then be potentially provided with behavioral and pharmacological risk reduction in the peripartum period.

Limitations of this study include the lack of data on lifestyle factors in the peripartum period, including breastfeeding, diet, and physical activity, as well as social determinants, which may modify the relationship between dysglycemia and ASCVD risk [13]. Strengths of this study include comprehensive assessment of dysglycemia in pregnancy and objective assessment of measures of ASCVD risk 10–14 years after delivery.

In conclusion, these results highlight the relationship between pregnancy dysglycemia, even without overt GDM, and long‐term ASCVD risk. This suggests that dysglycemia in the third trimester may identify those at increased risk of ASCVD after delivery with an opportunity to intervene years before actual disease manifests.

## CONFLICT OF INTEREST STATEMENT

The authors declare no conflicts of interest.

## FUNDING INFORMATION

The Hyperglycemia and Adverse Pregnancy Outcome Follow‐Up Study (HAPO FUS) was conducted by the HAPO FUS Investigators and supported by the National Institute of Diabetes and Digestive and Kidney Diseases (NIDDK) and the American Diabetes Association. The HAPO Study was supported by the National Institute of Child Health and Human Development and American Diabetes Association. The data from the HAPO FUS reported here were supplied by the NIDDK Central Repository. This manuscript was prepared in collaboration with some of the investigators of the HAPO FUS and does not necessarily reflect the opinions or views of the HAPO FUS, the NIDDK Central Repository, or the NIDDK. Dr. Venkatesh was supported by the Care Innovation and Community Improvement Program at The Ohio State University. Dr Khan was supported by NHLBI grant #HL161514. Dr. Shah was supported by American Heart Association grant #24CDA1266732.

## Data Availability

The data that support the findings of this study are openly available in NIDDK‐CR at https://repository.niddk.nih.gov/home/.
